# Lung injury does not aggravate mechanical ventilation-induced early cerebral inflammation or apoptosis in an animal model

**DOI:** 10.1371/journal.pone.0202131

**Published:** 2018-08-09

**Authors:** Jens Kamuf, Andreas Garcia-Bardon, Alexander Ziebart, Rainer Thomas, Konstantin Folkert, Katrin Frauenknecht, Serge C. Thal, Erik K. Hartmann

**Affiliations:** 1 Department of Anesthesiology, University Medical Centre, Johannes Gutenberg-University Mainz, Mainz, Germany; 2 Institute of Neuropathology, University Medical Centre, Johannes Gutenberg-University Mainz, Mainz, Germany; University of Colorado Denver, UNITED STATES

## Abstract

**Introduction:**

The acute respiratory distress syndrome is not only associated with a high mortality, but also goes along with cognitive impairment in survivors. The cause for this cognitive impairment is still not clear. One possible mechanism could be cerebral inflammation as result of a “lung-brain-crosstalk”. Even mechanical ventilation itself can induce cerebral inflammation. We hypothesized, that an acute lung injury aggravates the cerebral inflammation induced by mechanical ventilation itself and leads to neuronal damage.

**Methods:**

After approval of the institutional and state animal care committee 20 pigs were randomized to one of three groups: lung injury by central venous injection of oleic acid (n = 8), lung injury by bronchoalveolar lavage in combination with one hour of injurious ventilation (n = 8) or control (n = 6). Brain tissue of four native animals from a different study served as native group. For six hours all animals were ventilated with a tidal volume of 7 ml kg^-1^ and a scheme for positive end-expiratory pressure and inspired oxygen fraction, which was adapted from the ARDS network tables. Afterwards the animals were killed and the brains were harvested for histological (number of neurons and microglia) and molecular biologic (TNFalpha, IL-1beta, and IL-6) examinations.

**Results:**

There was no difference in the number of neurons or microglia cells between the groups. TNFalpha was significantly higher in all groups compared to native (p < 0.05), IL-6 was only increased in the lavage group compared to native (p < 0.05), IL-1beta showed no difference between the groups.

**Discussion:**

With our data we can confirm earlier results, that mechanical ventilation itself seems to trigger cerebral inflammation. This is not aggravated by acute lung injury, at least not within the first 6 hours after onset. Nevertheless, it seems too early to dismiss the idea of lung-injury induced cerebral inflammation, as 6 hours might be just not enough time to see any profound effect.

## Introduction

The acute respiratory distress syndrome (ARDS) is not only associated with a high mortality, but leads to cognitive impairment in survivors up to one year after hospital discharge [1, 2]. The cognitive dysfunction is characterized by deficits in global cognition or executive function [3]. Acutely it presents very often as delir [4] and 3 months after hospital discharge survivors show cognition scores similar to those typically seen in patients with moderate traumatic brain injury or mild Alzheimer’s disease [3]. The cognitive decline also affects also the quality of live in these patients and their relatives [5].

The cause for this impairment is still not known and several factors could contribute to this or are under discussion. One factor could be hypoxia, which is common in patients with ARDS. Especially the hippocampus is thought to be susceptible for hypoxic insults [6]. A study by Heuer et al. induced acute lung injury by hydrochloric acid (HCl)-instillation in the lungs of anesthetized pigs and found a non-significant increase in hippocampal damage [7]. Other likely key factors are inflammation [8], oxidative stress [9] or activation of the autonomic nervous system [10]. If one of these effects alone is sufficient for cognitive decline or if there are additional effects remains to be elucidated. Even mechanical ventilation itself is was shown to induce hippocampal apoptotic pathways [10] and could be causative or involved in the cognitive decline.

The present study therefore examines neuronal injury and inflammation beyond the trigger of mechanical ventilation in two porcine models of ARDS. We hypothesize that already an early ARDS induces cerebral inflammation and neuronal apoptosis in a model-independent fashion.

## Materials and methods

The institutional and state animal care committee (Landesuntersuchungsamt Rheinland-Pfalz, Koblenz, Germany), which is responsible for ethical evaluation and surveillance of animal studies, approved this study and the other study (results not yet published), of which we used native tissue samples in this study (approval numbers G13-1-071, G14-1-077). This prospective-randomized, controlled animal study was conducted in accordance with the international guidelines for the care and use of laboratory animals. To minimize suffering, all described experiments were conducted under general anesthesia. The native animals of the other study were housed, fed and euthanized like the animals in the present study. This manuscript adheres to the applicable EQUATOR guidelines.

### Anesthesia and instrumentation

20 healthy male pigs (sus scrofa domestica, weight: 24–31 kg) were examined in the study. All animals were delivered sedated (ketamine 4 mg kg^-1^, azaperon 8 mg kg^-1^ intramuscular) by a local breeder. After establishing an intravenous line anesthesia was induced and maintained by propofol (8–12 mg kg^-1^ h^-1^) and fentanyl (0.1–0.2 mg kg^-1^ h^-1^). A single dose atracurium (0.5 mg kg^-1^) was administered to facilitate orotracheal intubation. Ventilation (Respirator: Engström Carestation^®^, GE Healthcare, Germany) was started in pressure-controlled mode with a tidal volume (V_t_) of 7 ml kg^-1^, positive end-expiratory pressure (PEEP) of 5 cmH_2_O, fraction of inspired oxygen (FiO_2_) of 0.4 and a variable respiratory rate to maintain normocapnia. In all animals a balanced electrolyte solution (Sterofundin iso, B. Braun, Germany) was continuously infused at a rate of 5 ml kg^-1^ h^-1^. Vascular catheters were placed ultrasound-guided: an arterial line, a pulse contour cardiac output catheter (PiCCO, Pulsion Medical Systems, Germany), a central venous line and a 7.5-French introducer for a pulmonary arterial catheter were inserted via femoral vascular access. Respiratory and extended hemodynamic parameters were recorded continuously (Datex S/5, GE Healthcare, Germany). Cardiac output and extravascular lung water index were measured by single-indicator transpulmonary thermodilution using the PiCCO system according to the manufacturer´s instructions. Each measurement was performed as triplicate. Further parameters were recorded by the respirator. Normothermia was maintained by body surface warming.

### Study protocol

Following instrumentation, we set the F_i_O_2_ to 1.0 and conducted a lung recruitment maneuver (plateau pressure 40 cmH_2_O for 10 seconds). Then baseline parameters were assessed at healthy state. Afterwards the pigs were randomized to one of three groups: lung injury by central venous injection of oleic acid (OAI; n = 8), lung injury by bronchoalveolar lavage (LAV; n = 8) or control (Ctr; n = 6). Brain tissue of 4 native animals served as native group (native, n = 4). For LAV the endotracheal tube was clamped in inspiration. Then 30 ml kg^-1^ of warmed balanced saline solution were instilled and directly drained by gravity. Lavage procedures were repeated within 10 min intervals until a PaO_2_/FiO_2_ ratio ≤ 250 mmHg was achieved. The lavage procedure was followed by a second hit of injurious ventilation for one hour: pressure-controlled mode, *V*_t_ of 15 ml kg^-1^, PEEP 0, FiO_2_ 1.0, and respiratory rate to maintain normocapnia. For OAI 0.1 ml kg^-1^ of oleic acid (cis-9-octadecenoic acid) were solved in 20 ml saline solution and injected via the central venous line in fractions of 2 ml every three minutes. The procedure was repeated with another 0.1 ml kg^-1^ after 15 minutes, if the PaO_2_/FiO_2_ was higher than 200 mmHg.

After ARDS criteria were achieved by OAI respectively LAV in combination with one hour of injurious ventilation the animals were treated according to a standard protocol, which was closely adapted to the ARDS Network low PEEP table. The respirator settings were: *V*_t_ 7 ml kg^-1^, FiO_2_ and PEEP according to the dedicated table with an intended peripheral oxygen saturation (SpO_2_) of 94–98%. The Ctr animals were ventilated with the same protocol. If necessary to warrant stable hemodynamics during the experiments (mean arterial pressure > 65 mmHg), norepinephrine was administered. After 6 hours the animals were killed in deep general anesthesia by injection of 200 mg propofol and 40 mmol potassium.

### Post-mortem analysis

After death, brain and lungs were harvested for further investigations. The brain was cut in two halves, one halve was fixated in 4% formaldehyde solution, from the other halve we took a slice of the frontal cortex and the hippocampus [11]. Both samples were snap frozen in liquid nitrogen for molecular biological analysis.

Initially the hippocampus was cut in 2 mm thick slices. 4 of those slices were then cut rostro caudal and embedded in paraffine. Afterwards they were cut further in 4 μm thick histological preparations. These preparations were hematoxylin/eosin (HE) or Iba-1 stained and examined with light microscope at 20x zoom for differences in the number of neurons or microglia cells. Therefore, parts of cornu ammonis (CA1 and CA2) were chosen as regions of interest and a 1950 x 1936 pixel large picture was examined [12].

The left lung was weighted immediately after removal and dried afterwards at 60°C for 72 hours to determine the dry weight and wet-to-dry ratio. Histopathologic assessment of lung injury was performed with an established score with a maximum of 175 points [13]. Samples from the non-dependent, central, and dependent lung regions were fixed in formalin. Then the samples were embedded in paraffin and HE stained. Seven histopathological facets of injury were examined by a blinded investigator: alveolar edema, interstitial edema, hemorrhage, epithelial destruction, inflammatory infiltration, overdistension, microatelectasis. In each lung region the seven markers were ranked from zero to five points in four non-overlapping microscopy fields as well as in a global overview of the region [13]. To determine cerebral production of mRNA of IL-6, IL-1beta and TNFalpha we used realtime-PCR (Lightcycler 480 PCR System, Roche Applied Science, Germany) like published before [14].

### Statistics

Data are reported as mean and standard deviation. Statistical analyses were performed by One-way ANOVA with post-hoc tests for multiple testing (Holm-Sidak). The results were analyzed and graphed using Sigmaplot^®^ 12.5. A p < 0.05 was considered significant.

## Results

A total of 20 animal experiments were included in this study, brain tissue of 4 native animals served as native group. Lung injury led to sustained gas exchange impairment, increase of the extracellular lung water content, pulmonary arterial hypertension and histologic traceable lung injury. The physiologic parameters are summarized in Table 1.

**Table 1:** Physiologic parameters given as mean ± SD.

		OAI	LAV	Ctr
PaO_2_/FiO_2_ [mmHg]	Baseline	504±150	448±41	462±34
	0h	102±36*#	162±49*	595±36
	3h	184±78*	200±60*	445±20
	6h	241±145*	231±42*	426±48
etCO_2_ [mmHg]	Baseline	39±3	40±2	40±1
	0h	43±3*	40±2	37±4
	3h	42±4	39±3	39±3
	6h	43±4	39±4	38±3
V_t_ [ml/kg]	Baseline	7.0±0.5	7.2±0.5	7.2±0.3
	0h	6.8±0.4	7.3±0.5	7.4±0.4
	3h	7.1±0.4	7.3±0.3	7.2±0.4
	6h	7.2±0.5	7.2±0.4	7.1±0.3
Ppeak [cm H_2_O]	Baseline	16±3	16±1	16±3
	0h	28±5*	28±4*	15±2
	3h	30±5*	27±4*	15±2
	6h	29±5*	27±4*	16±2
Pmean [cm H_2_O]	Baseline	8±1	9±2	8±1
	0h	12±2	14±6*	8±1
	3h	16±2*	14±5*	8±1
	6h	16±2*	13±4*	8±1
PEEP [cm H_2_O]	Baseline	5±0	5±1	5±0
	0h	6±2	6±1	5±0
	3h	10±2*#	7±2*	5±0
	6h	11±2*#	7±1	5±0
MAP [mmHg]	Baseline	80±15	76±14	78±7
	0h	75±4	78±7	79±7
	3h	73±5	76±9	72±5
	6h	69±4	78±11	73±9
MPAP [mmHg]	Baseline	15±3	17±3	17±4
	0h	36±2*#	27±5*	15±3
	3h	28±5*	27±10*	15±3
	6h	26±5*	22±3	17±4
CO [l/min]	Baseline	3.3±0.7	3.7±0.7	3.5±0.5
	0h	3.3±0.6#	4.4±0.7	3.6±0.9
	3h	3.2±0.5#	4.6±1.0	3.6±0.7
	6h	3.4±0.4#	4.2±0.6	3.5±0.7
EVLWI [ml/kg]	Baseline	10±1	10±1	11±2
	0h	22±7*#	15±1	12±2
	3h	22±10*#	14±2	13±2
	6h	22±7*#	14±4	12±1

* p < 0.05 vs Ctr

^#^ p < 0.05 vs. LAV

(Abbreviations: etCO_2_ = endtidal carbon dioxide; V_t_ = tidal volume; Ppeak = airway peak pressure; Pmean = airway mean pressure; PEEP = positive endexpiratory pressure; MAP = mean arterial pressure; MPAP = mean pulmonary arterial pressure; CO = cardiac output; EVLWI = extravascular lung water index)

**Fig 1:**
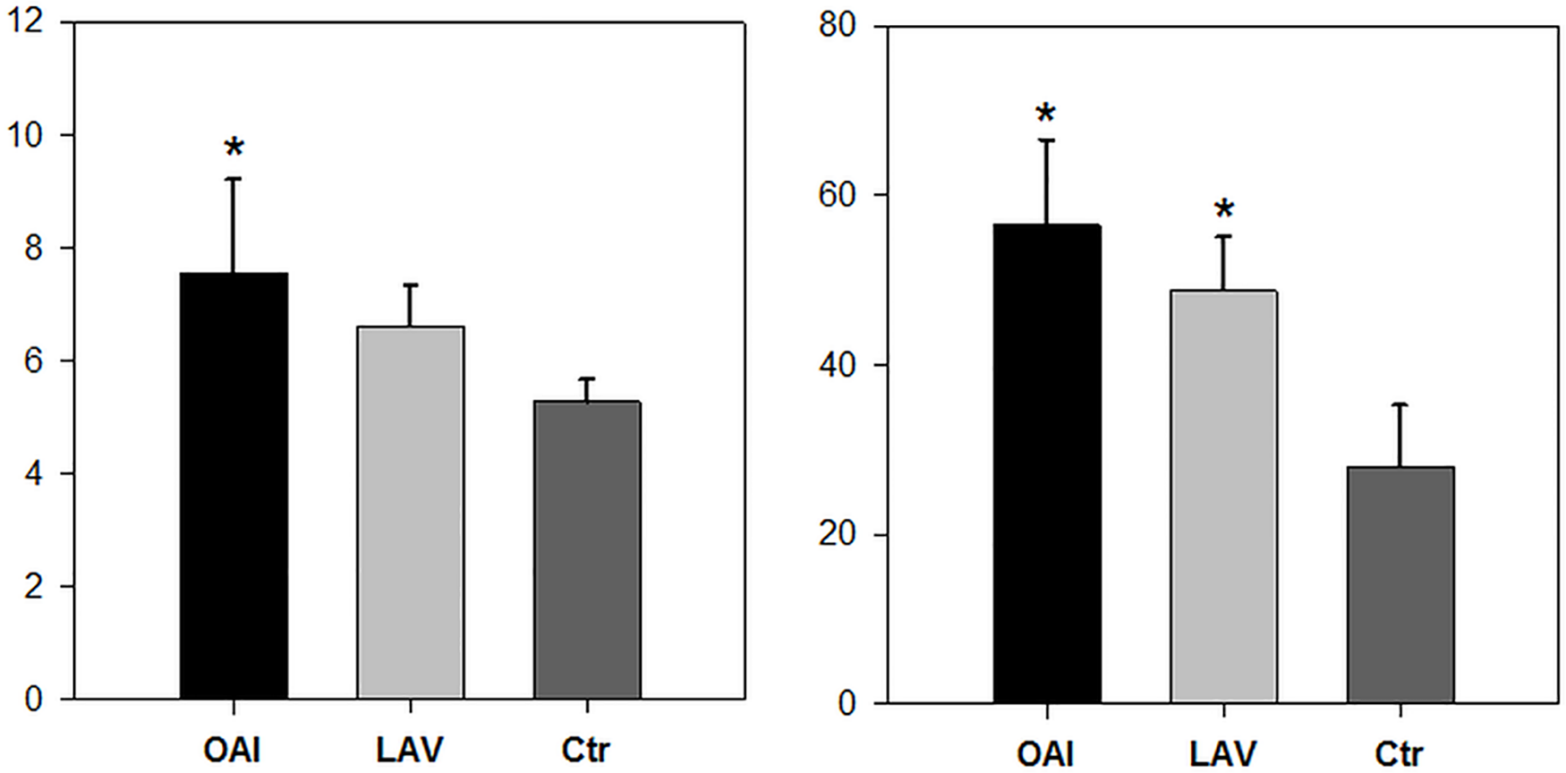
Wet-to-dry ratio and lung injury score. Wet-to-dry ratio (left) and histologic assessed lung damage score (right). * p < 0.05 vs. Ctr.

As indicator for pulmonary edema we assessed the pulmonary wet-to-dry ratio. We found a significant increase in the pulmonary edema of the animals of the OAI group compared to Ctr group (p = 0.004), there was no significant difference between the animals of the LAV compared to the Ctr group or the OAI and the LAV group (Fig 1). Histologic examination of the lung samples yielded significant increase in the lung damage score in the animals of the OAI and LAV group compared to Ctr (each p < 0.001). There was no difference between the OAI and LAV group (Fig 1).

**Fig 2:**
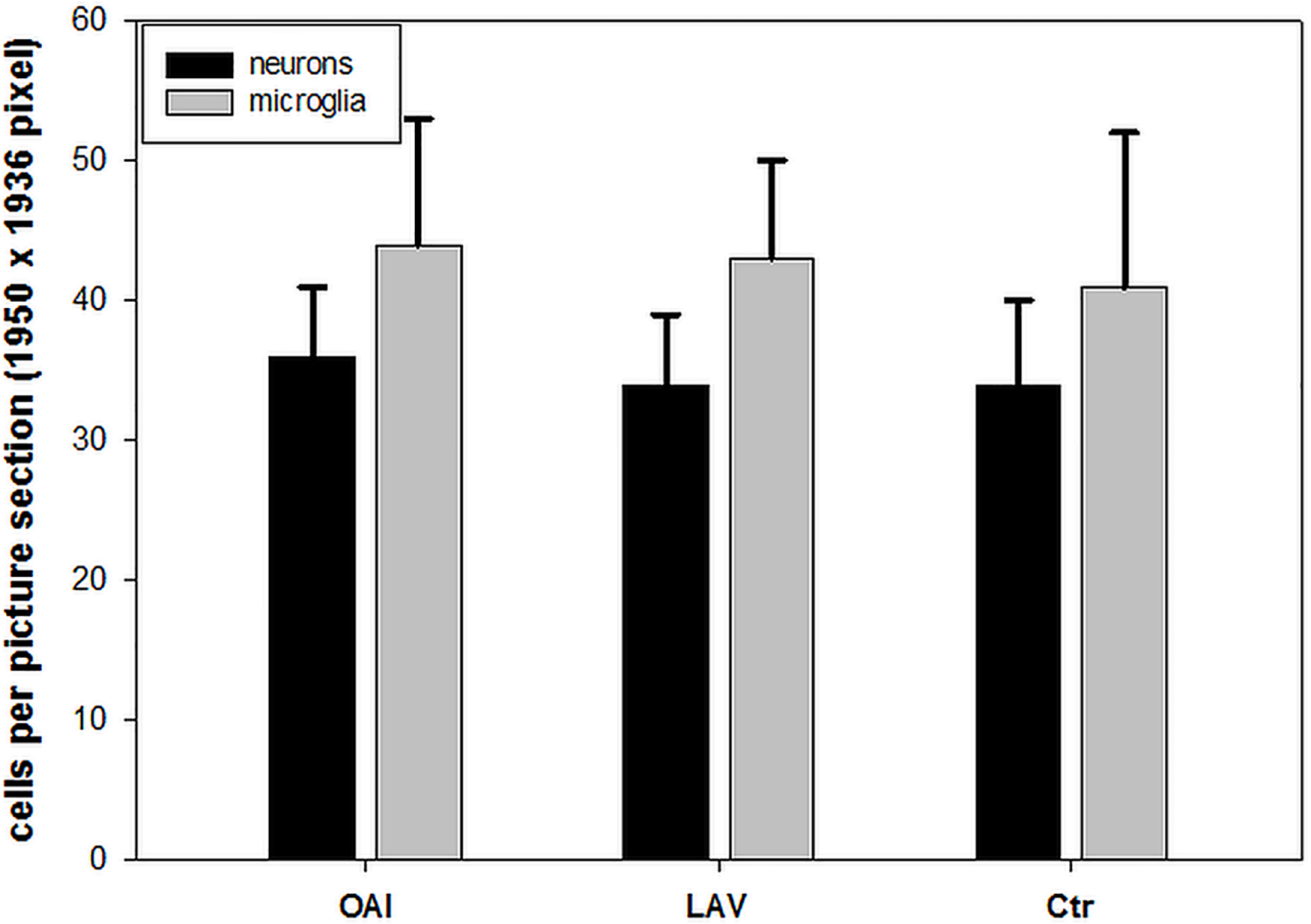
Histology brain. Number of HE-stained neurons and Iba-1-stained microglia cells.

For histologic assessment of cerebral inflammation and apoptosis we examined the number of neutrons in HE-stained slices and the number of Iba-1-positive cells. There was no difference regarding the number of HE-stained neurons between the groups (OAI 35.6 ± 4.9, BAL 33.4 ± 6.8, Ctr 33.7 ± 5.7). The microscopic evaluation of Iba-1-stained brain slices showed in the OAI group 43.5 ± 9.4 leucocytes, in the LAV group 43.4 ± 7.0 leucocytes and in the Ctr group 40.5 ± 11.2 leucocytes, which means there is no difference in between the groups (Fig 2).

**Fig 3:**
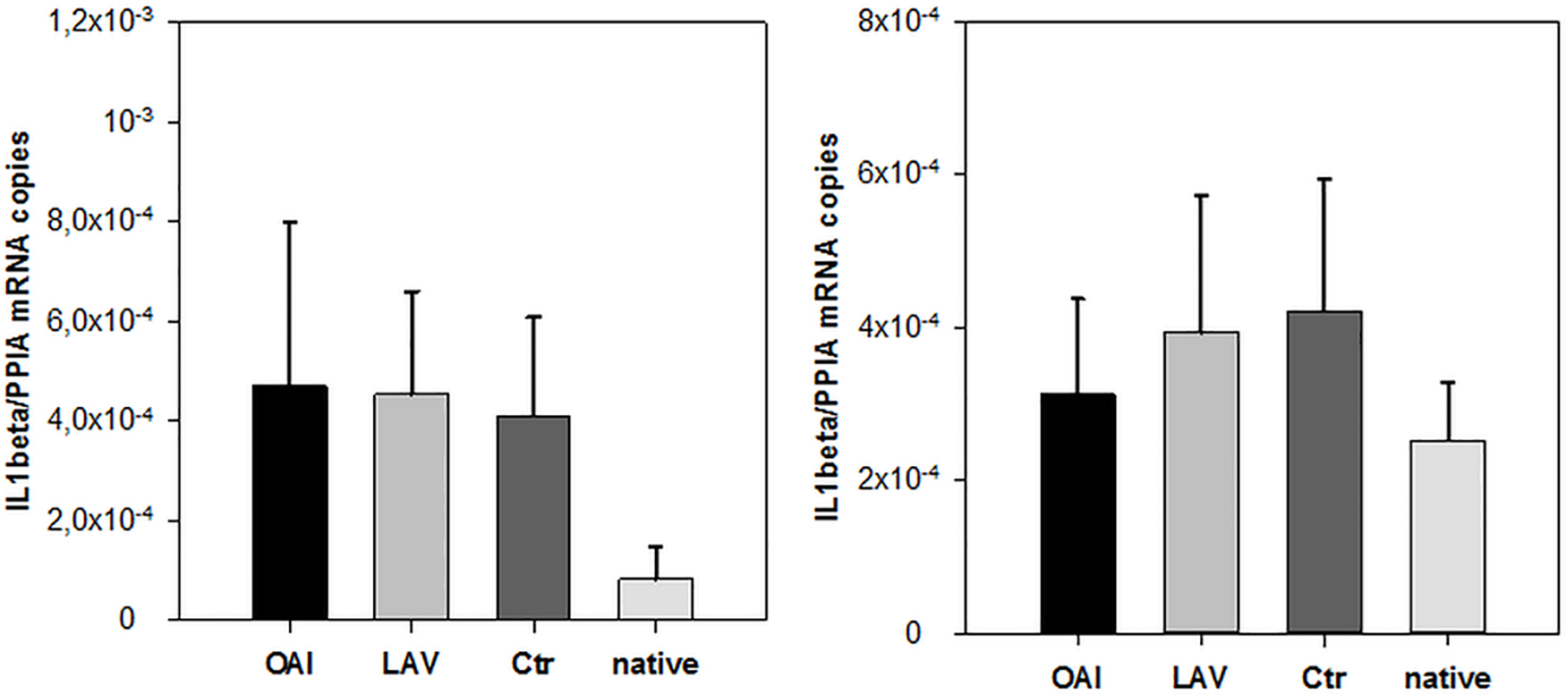
IL-1beta in cortex or hippocampus. IL-1beta mRNA in cortex (left) or hippocampus (right).

**Fig 4:**
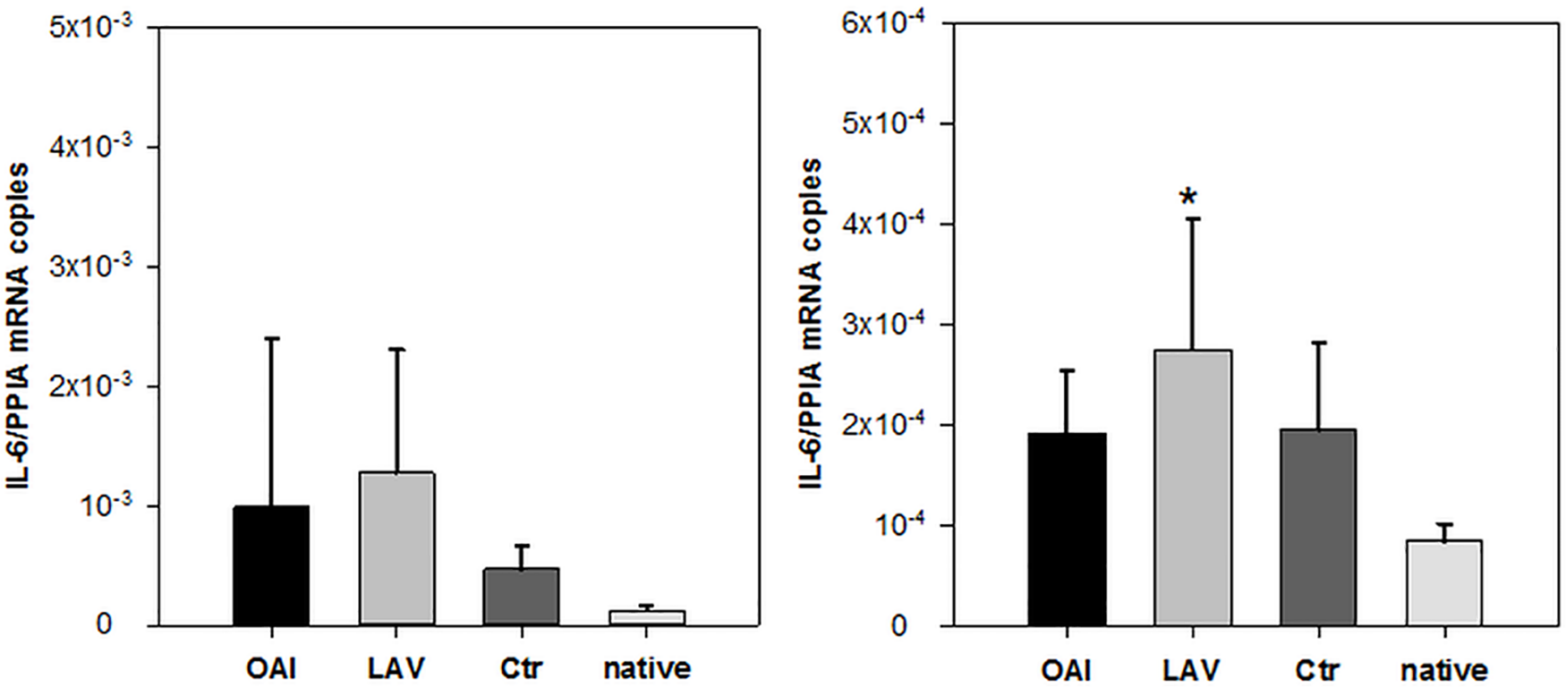
IL-6 in cortex or hippocampus. IL-6 mRNA in cortex (left) or hippocampus (right). * p < 0.05 vs. Ctr.

**Fig 5:**
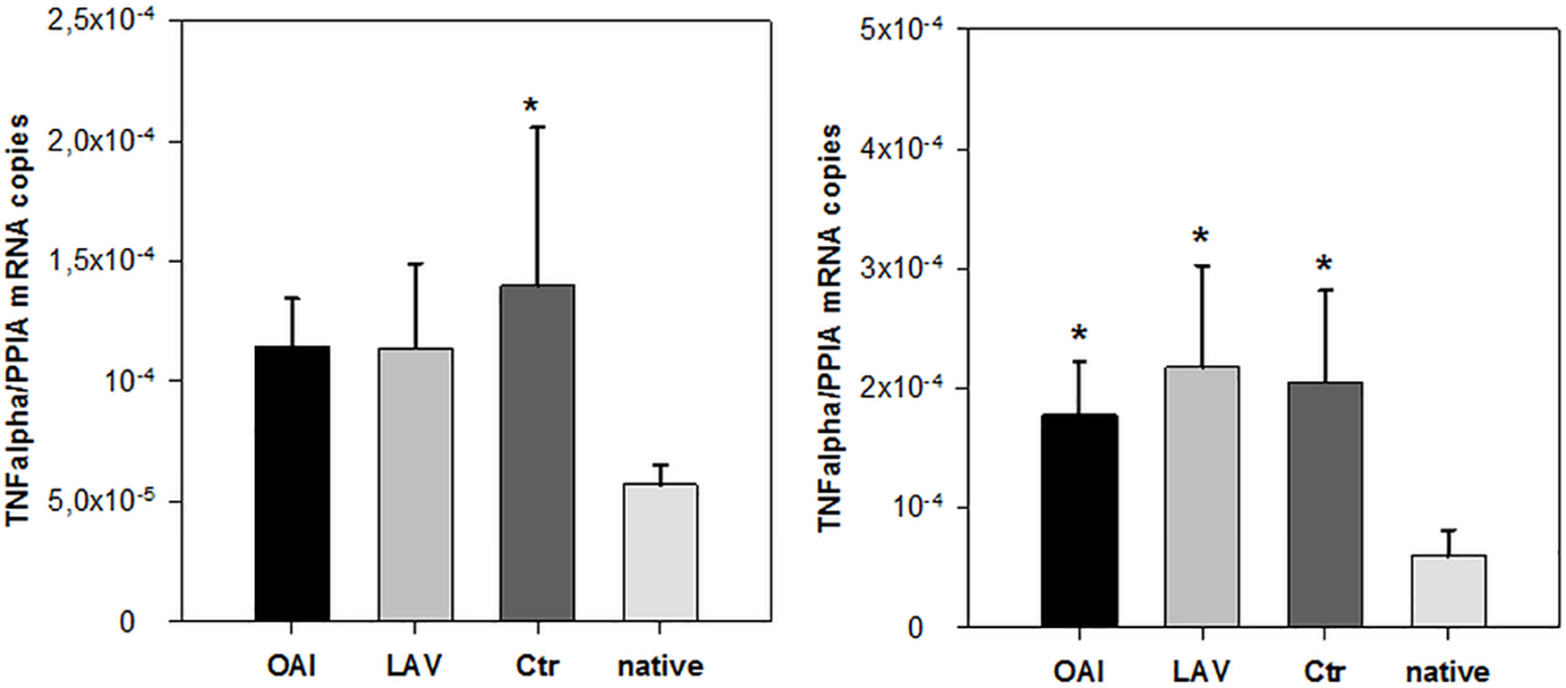
TNFalpha in cortex or hippocampus. TNFalpha mRNA in cortex (left) or hippocampus (right). * p < 0.05 vs. native.

Regarding the cytokine IL-1beta we found no difference in the number of RNA-copies normalized to peptidylprolyl isomerase A (PPIA) in the cortex or hippocampus samples (Fig 3). IL-6 expression did not differ in the cortex samples (Fig 4). There was a significantly higher IL-6 expression in the hippocampus samples of the LAV group compared to native tissue (p = 0.02; Fig 4), but no other difference between any groups. TNFalpha was significantly increased in the hippocampus of all three groups receiving ventilation in comparison to the native group (LAV vs. native p = 0.004; OAI vs. native p = 0.029; Ctr vs. native p = 0.011; Fig 5). In the cortex TNFalpha expression showed a similar overall trend (p = 0.028), which only became significant in the Ctr group after the post-hoc correction (p = 0.02; Fig 5).

## Discussion

The acute respiratory distress syndrome is a complex syndrome with different pathologies [15]. To date there is no single model to fully reflect the pathophysiologic changes in patients suffering from ARDS, but several models simulate different aspects of this syndrome [16]. In human ARDS inflammatory pathways play a central role [17, 18] and lung-derived circulating mediators possibly cause multiorgan failure in critically ill patients [19]. Even mechanical ventilation itself seems to have neuro-detrimental properties as well [10]. In the present study we wanted to examine, if early lung injury aggravates the ventilator-induced neuronal damage. Therefore, we compared cerebral inflammation and damage in mechanically ventilated pigs with and without ARDS. We chose two different lung injury models with different properties: central venous injection of oleic acid and bronchoalveolar lavage followed by one hour of injurious ventilation. Oleic acid is the most abundant fatty acid in healthy humans. It directly affects the lung and triggers activation of innate immune receptors [20]. This leads to neutrophil accumulation [21], inflammatory mediator production [22], and cell death [20]. Oleic acid-induced lung injury is a well-established model that has been used in different animals with reproducible results [16, 23]. The combination of bronchoalveolar lavage with high tidal volume ventilation is a so-called “two hit model”. The lavage procedure leads to surfactant depletion, which increases alveolar surface tension and facilitates alveolar collapse [22]. This can also reduce the alveolar host defense as surfactant protein-A is thought to be protective against an overly activated immune system [24]. Surfactant depletion alone leads to only minimal functional damage of the lungs, but makes the lung more susceptible for ventilator-induced lung injury [16, 23]. In our study, we found hints for histologic lung injury in both models, a drop in PaO_2_/FiO_2_ ratio and an increase of the pulmonary arterial pressure. This is in accordance with other publications [25, 26]. The wet-to-dry ratio and EVLWI were markedly higher in the OAI group than in the LAV group indicating a less severe barrier dysfunction through LAV. This is consistent with other studies comparing those models [27]. In both groups pulmonary arterial pressure as well as PaO_2_/FiO_2_ ratio improved during the course of the study, maybe as result of the continuous lung protective ventilation with moderate PEEP and low tidal volumes.

The central element of our study was neuronal inflammation and damage. Thereby we focused on the absolute number of neurons as indicator for neuronal damage, Iba-1-dyed microglia cells for immune activation and mRNA expression of IL-6, TNFalpha and IL-1beta as indicators of cerebral cytokine production. Microscopy of hippocampus slices showed no difference in the number of HE-dyed neurons between the ventilated animals with or without lung injury or brain tissue from native animals. This confirms the results of Bickenbach et al. [28], who found no hints for early neuronal damage in mechanically ventilated pigs that underwent HCl-induced lung injury or hypoxemia. In another study pulmonary HCl instillation in pigs caused a non-significant occurrence of hippocampal damage assessed by a decrease of neurons counts [7]. In this study intracranial pressure was measured invasively in all animals, so the authors concluded that this procedure induced a baseline damage, which reduced the difference between the groups and hampered statistically significant findings. Our results are in contrast to a study in non-hypoxemic mice, which received either low-pressure ventilation with a peak pressure of 12 cmH_2_O and a PEEP of 2 cmH_2_0 or high-pressure ventilation with a peak pressure of 20 cmH_2_O and a PEEP of 0 cmH_2_O for 90 minutes [10]. Both groups showed an increase of pro-apoptotic markers compared to non-ventilated control animals. Inflammatory markers were not different between the groups. These differences can be explained by different experimental techniques. Gonzales-Lopez et al. [10] examined pro-apoptotic markers, whereas our study focused on actual numbers of neurons. As an increase in pro-apoptotic markers precedes the death of cells, the period between insult, activation of apoptosis and degradation of cells with countable difference in the number of neurons may be too short.

Microglia are the main active immune cells in the central nervous system. They can persist in a resting or an activated state [29]. In the resting state they screen their environment for potential threats. Detection of danger signals leads to their activation with subsequent differentiation, migration and cytokine production. There is no data about microglia activation in ARDS. But sepsis has been associated with activation of microglia [30], which is related with delirium, neurodegenerative deficits, and potentially persisting neurotoxic effects [31]. In our study, we found no relevant differences in the number of Iba-1-dyed microglia. Nevertheless, it seems too early to dismiss the hypothesis of ARDS-induced activation of cerebral microglia. Even under septic conditions the period between the activating stimulus and cerebral activation of microglia cells varies from few hours up to several days [32], so maybe a longer time span is necessary for pronounced effects.

Activated microglia not only migrate and proliferate, but also produce cytokines like TNFalpha, IL-1beta and IL-6 [32]. None of these cytokines showed any difference between the ventilation only and the lung injury groups, whereas there was a considerable difference between the native animals and all experimental groups regarding TNFalpha mRNA expression. IL-6 was only increased in the animals of the LAV group compared to native animals. In a study concerning cerebral inflammation through mechanical ventilation by itself anesthetized mice were either mechanically ventilated for six hours or spontaneously breathing: this study yielded elevated hippocampal amounts of IL-6, TNFalpha, and IL-1beta in the mechanically ventilated group, which was paralleled by increased systemic cytokine levels [33]. As these cytokines are able to cross the blood brain barrier [34], the origin of the cytokine increase, which was assessed by their total protein content, remains unclear. In contrary, we examined the cerebral mRNA expression of these cytokines, which may serve as another explanation for the contrary findings.

Our study also has some limitations. We didn’t randomize animals to the native group, but used native tissue obtained in another study. This is reasonable in terms of reducing animal experiments, but leads to missing data from the native group concerning histopathologic examinations. As our focus was to identify potential differences between ventilated animals with or without lung injury, this approach seems acceptable. Another limitation is the mere examination of cytokines by realtime-PCR without using other methods like Western Blots, which would have improved the quality of our study. Furthermore, our study may be underpowered due to its explorative character.

## Conclusion

To our knowledge, this is the first study focusing on the neuroinflammatory and -damaging potential of early ARDS. We found hints of an increased cerebral expression of inflammatory cytokines within six hours after onset of lung injury, but no signs of histopathologic injury. Our data do not allow for the conclusion that early cerebral inflammation occurs beyond the effect of mechanical ventilation. Nevertheless, it may be too early to dismiss the concept of lung injury-induced brain dysfunction or inflammation. Patients suffering from ARDS usually need several days of intensive care treatment. Therefore, it may be helpful to extend the duration of the experiments.
